# A new model using deep learning to predict recurrence after surgical resection of lung adenocarcinoma

**DOI:** 10.1038/s41598-024-56867-9

**Published:** 2024-03-16

**Authors:** Pil-Jong Kim, Hee Sang Hwang, Gyuheon Choi, Hyun-Jung Sung, Bokyung Ahn, Ji-Su Uh, Shinkyo Yoon, Deokhoon Kim, Sung-Min Chun, Se Jin Jang, Heounjeong Go

**Affiliations:** 1 School of Dentistry and Dental Research Institute, Seoul National University, Seoul, Republic of Korea; 2 Department of Pathology, Asan Medical Center, University of Ulsan College of Medicine, Seoul, Republic of Korea; 3 Department of Oncology, Asan Medical Center, University of Ulsan College of Medicine, Seoul, Republic of Korea

**Keywords:** Deep learning, Lung adenocarcinoma, Recurrence, Histopathology, Pathology image, Prognostic markers, Lung cancer

## Abstract

This study aimed to develop a deep learning (DL) model for predicting the recurrence risk of lung adenocarcinoma (LUAD) based on its histopathological features. Clinicopathological data and whole slide images from 164 LUAD cases were collected and used to train DL models with an ImageNet pre-trained efficientnet-b2 architecture, densenet201, and resnet152. The models were trained to classify each image patch into high-risk or low-risk groups, and the case-level result was determined by multiple instance learning with final FC layer’s features from a model from all patches. Analysis of the clinicopathological and genetic characteristics of the model-based risk group was performed. For predicting recurrence, the model had an area under the curve score of 0.763 with 0.750, 0.633 and 0.680 of sensitivity, specificity, and accuracy in the test set, respectively. High-risk cases for recurrence predicted by the model (HR group) were significantly associated with shorter recurrence-free survival and a higher stage (both, *p* < 0.001). The HR group was associated with specific histopathological features such as poorly differentiated components, complex glandular pattern components, tumor spread through air spaces, and a higher grade. In the HR group, pleural invasion, necrosis, and lymphatic invasion were more frequent, and the size of the invasion was larger (all, *p* < 0.001). Several genetic mutations, including *TP53* (*p* = 0.007) mutations, were more frequently found in the HR group. The results of stages I-II were similar to those of the general cohort. DL-based model can predict the recurrence risk of LUAD and identify the presence of the *TP53* gene mutation by analyzing histopathologic features.

## Introduction

Lung cancer is the leading cause of cancer morbidity and mortality worldwide, and the incidence of lung adenocarcinoma (LUAD) is still increasing^1,2^. Currently, locoregional treatment such as surgical resection or radiation therapy is recommended as standard treatment in stages I–II LUAD, except for some cases of stage IIB showing invasive growth^3^. However, postoperative recurrence is frequent even after complete resection of lung cancer, and the prognosis is generally poor even with salvage treatment^4^. Therefore, predicting the risk of recurrence of lung cancer patients would be very useful when selecting the adjuvant treatment plan.

One of the key factors correlated with recurrence is tumor histology. Of note, a new international association for the study of lung cancer (IASLC) grading system for invasive LUAD has been validated with improved recurrence-free and overall survival discrimination. Tumor spread through air spaces (STAS), a novel invasive pattern of non-small cell lung cancer (NSCLC), has been demonstrated in many studies to be strongly correlated with recurrence after resection, especially in stage I cancers^5,6^ but the concept has been criticized because of the difficulty to discriminate the artifacts associated with specimen handling^7^. In addition, various histopathologic features, such as pathologic TNM stage, tumor size, solid and micropapillary patterns, resection margin status, invasion of blood vessels and/or pleura, and tumor microenvironment have a significant correlation with patient prognosis^8^. However, a detailed histopathologic examination of lung cancer is very difficult and laborious, making it vulnerable to error. According to the results of a previous study, the reproducibility of the current IASLC grading system is good, but not very high, even for expert pathologists^9^.

Recent advances in digital pathology could help solve this problem. Developments in machine learning (ML)-based image analysis techniques, especially in deep learning (DL), have shown that they can assist with diagnoses, identify novel features, and predict patients’ outcomes^10^. Research into ML-based histological analysis of lung cancer has mainly dealt with segmentation of tumor boundaries and the classification of tumor types^11–13^. Several studies tried to predict patient outcomes by automatic histological analyses of histomorphometric features^14,15^ and tumor microenvironment features^16^. Recent studies showed that DL-based analysis of images, not histomorphometric features, could predict the recurrence of LUAD^17,18^. They had meaningful predictive performance, but they lacked analyses about the relationship between the models’ output and other histopathologic parameters^19^.

In this study, we aimed to develop a new DL-based model to predict the recurrence of LUAD, and then we investigated the results in the context of histopathological parameters and tumoral genetic aberrations.

## Materials and methods

### Clinicopathological data acquisition

**Figure 1:**
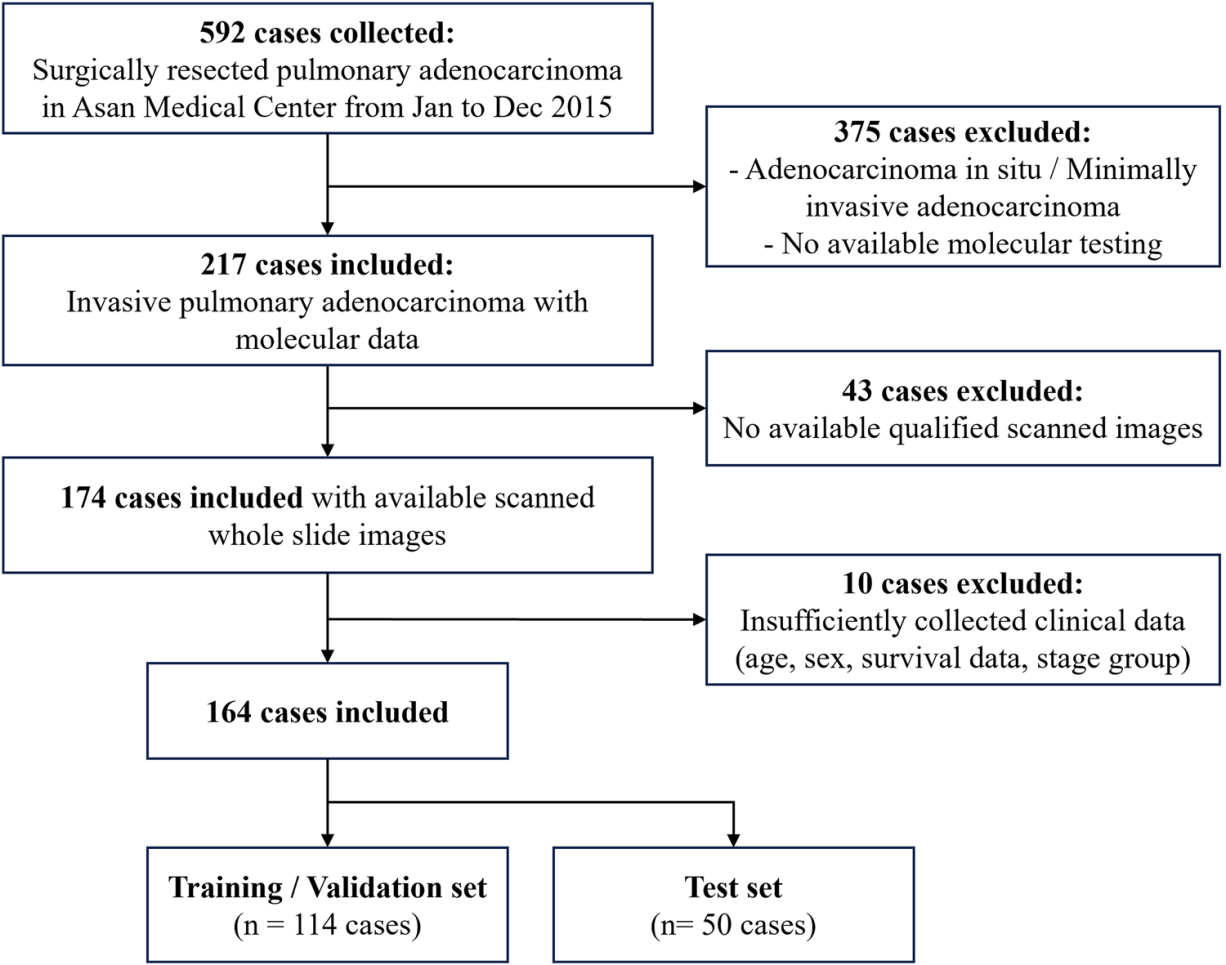
Flowchart of inclusion and exclusion of the patient cohort.

Clinical, pathological, and genomic data were retrieved from a previously reported cohort^20^. It consists of 164 cases of lung adenocarcinoma that were surgically resected from January 2015 to December 2015. Their data were retrospectively retrieved at Asan Medical Center (AMC), Seoul, Republic of Korea^20,21^. The pathological data were reviewed by pulmonary pathologists (HSH and BA). Patients’ pathological diagnoses were established in line with the World Health Organization (WHO) criteria^8^, IASLC guideline^9^ and the 8th edition of the American Joint Committee on Cancer (AJCC) Cancer Staging Manual^22^. Tumor samples were subjected to targeted next-generation sequencing (NGS) using the AMC OncoPanel version 4, a custom cancer panel encompassing the entire exome area or mutation hotspot regions of 334 cancer-related genes and intron area of fusion hotspots of the *ALK, EGFR, NTRK1, RET, ROS1,* and *BRAF* genes^20^. The inclusion and exclusion criteria for patients are summarized in Fig. 1.

### Image data preparation for training the deep learning model

One representative hematoxylin & eosin (H&E)-stained slide was selected from each case by manual review blinded to clinical and pathological information. The slides were scanned with a 3D Histech Panoramic 250 Flash II (Budapest, Hungary) scanner at 20× magnification and a resolution of 0.221 µm per pixel. Whole slide images (WSIs) were exported in mrxs format. Four expert pathologists (GC, HJS, JSU, and HG) annotated the boundaries of the tumor site using QuPath 0.3.0 (https://qupath.github.io). It was reconfirmed in all images that the annotation results correctly indicated the tumor site.

For developing the DL model, image patches (256 × 256 pixels) were randomly extracted from the annotated tumor area with an average of 100 patches per non-recurrent case and 148 patches per recurrent case to balance the data size between the recurrent and non-recurrent groups. In total, 19,188 patches were retrieved. They were randomly divided into independent training and test sets at a ratio of 7:3. Cross validation method with fivefold was involved in train procedure. The images were normalized with the training set data in each channel.

### Training method of the deep learning model

Due to the complexity and small size of this study’s data set, a lightweight network with fewer parameters was suitable because it requires less training time and achieves a performance comparable to other networks. To decide suitable DL model, we compared efficientnet-b2, densenet201 and resnet152. After comparing these DL modeling’s accuracy metrics in cross validation, we chose efficientnet-b2 architecture as our classifier, considering its special design for improving accuracy and efficiency through AutoML and model scaling with a verified ability to accomplish classification tasks with high accuracy while using a relatively small number of parameters (~ 7 million)^23^. The model network used ImageNet based pre-trained initialization of weights and was trained with cross-entropy as the loss function. The model parameters were updated by Adam optimizer with 0.9 β_1_ and 0.999 β_2_^24^. The network was trained with a batch size of 256 and an initial learning rate of 1e−6. The model parameters were iteratively updated to decrease the cross entropy. The model was saved when the least loss of cross-entropy was obtained in the validation set and then it was used for further evaluation and manipulation.

The input data were individual tumor image patches. Ground truth was the status of tumor recurrence of the case from which the image patch was extracted. During model training, data augmentation was applied to improve its robustness: flipping, translation, rotation, and color augmentations, including random contrast (multiplication by 0.5–1.5), brightness (multiplication by 0.65–1.35), hue (addition by − 32 to 32) and value (addition by − 32 to 32). The DL network was developed with the PyTorch framework (version 1.11.0) on a dual NVIDIA GeForce RTX 3090 under the Python (version 3.8) environment.

### Performance evaluation of the model

The model classified each image patch into low-risk (LR) or high-risk (HR) groups according to the output (the model-based feature). Image patches classified as the HR group and extracted from a case with actual tumor recurrence were considered true positive, and vice versa. A case-level output was determined by multiple instance learning with 2-layer preNN and 1-layer afterNN with final FC layer’s 1408 features from a model from all patches^25^.

The average value of the model-based features of the extracted patches. A confusion matrix was used to illustrate the performance of the trained model on the training, validation, and testing set with 4 categorical results [true positives (TP), true negatives (TN), false positives (FP), and false negatives (FN)]. Besides, additional parameters, including sensitivity, specificity, positive predictive value (PPV), negative predictive value (NPV) and F1-score were calculated to obtain a comprehensive performance measure of the results. The 95% confidence intervals (CIs) of sensitivity, specificity, PPV and NPV were calculated to estimate the corresponding variability^26^. To validate its clinical performance, recurrence-free survival (RFS) rates by risk group were compared using the Kaplan–Meier method and the log-rank test.

### Clinicopathological analysis and statistical methods

We analyzed the associations between the model-based results at the case-level and the pathological parameters. Whole data (164 cases) were used in this analysis because this was not for validating the model’s performance but for acquiring insights into the model’s interpretation. The proportion of poorly differentiated (PD) components and complex glandular pattern (CGP) were evaluated by eyeballing by expert pulmonary pathologists. PD components include solid, micropapillary, cribriform, and CGP. CGP include fused glands with irregular borders and single cells infiltrating desmoplastic patterns^9^. Differences between continuous variables in two groups were evaluated by Student's t-test. Differences in frequencies of categorical variables were estimated by a chi-square test with correction. All statistical evaluations were performed with R version 4.2.1 (The R Foundation for Statistical Computing, Vienna, Austria). *p* value < 0.05 was considered statistically significant.

### Ethical approval

This study was conducted according to the ethical guidelines of the Declaration of Helsinki. All studies involving patients were examined and approved by the Institutional Review Board of Asan Medical Center (IRB approval number: 2018-1198). The requirement for written informed consent was waived by IRB of Asan Medical Center because of the retrospective nature of the study and use of deidentified data.

## Results

### Risk prediction performance of the model

**Figure 2:**
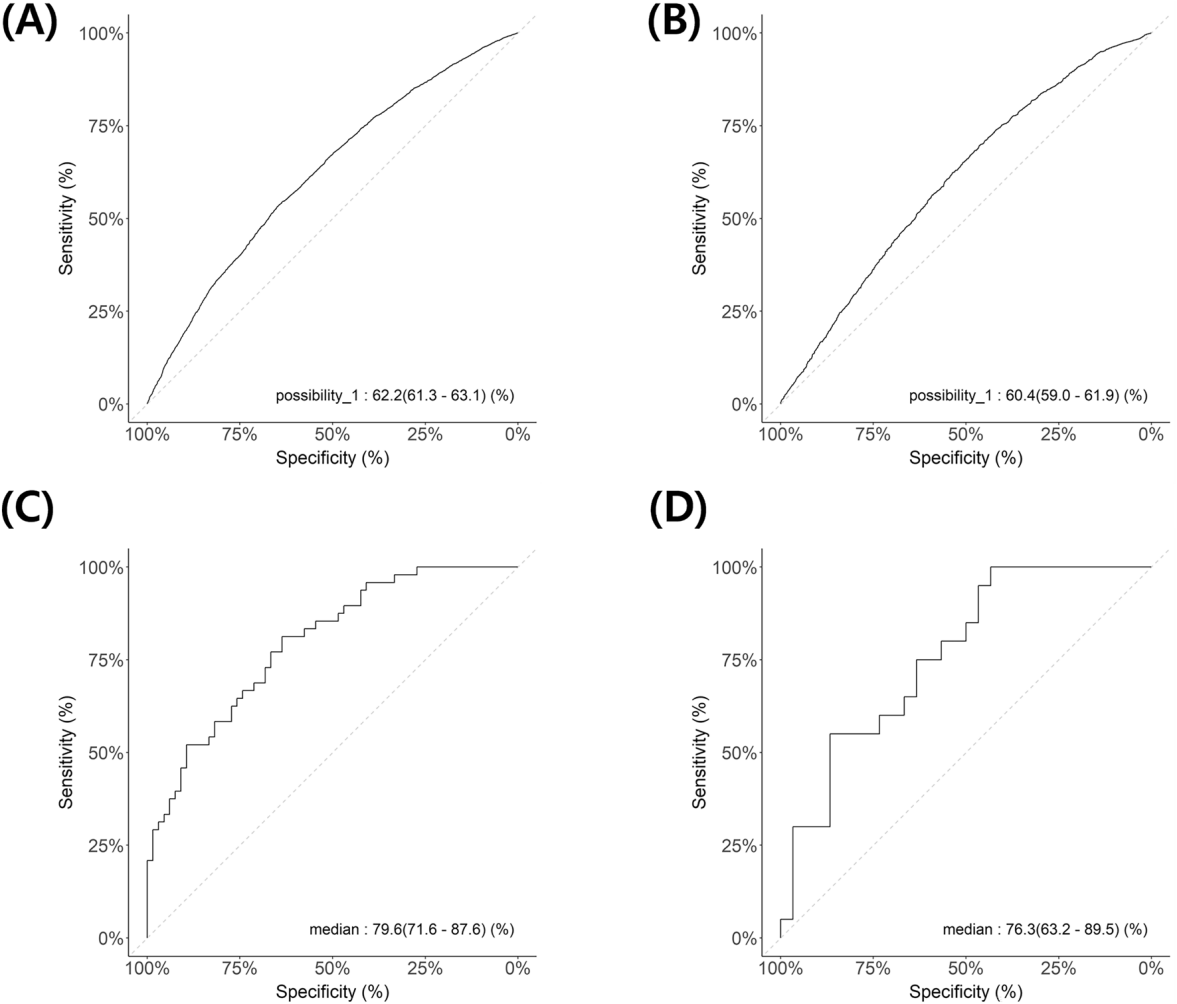
Receiver operating curves of the model at the patch-level (**A**–**C**, A: training set, B: validation set, C: testing set) and at the case-level (**D**–**F**, D: training set, E: validation set, F: testing set).

Efficientnet-b2, densenet201, and resnet152 were compared based on cross-validation accuracy at the patch level, and as a result, efficientnet-b2 was chosen as the final learning architecture (Supplementary Table S1). The model performance at the patch-level and case-level were summarized in Table 1. At the patch-level, the model achieved a sensitivity of 70.7% and a specificity of 46.0%. The F1 score was 0.6332 and the accuracy was 58.5%. The area under the curve (AUC) of the receiver operating curves (ROC) in the training, and test sets were 0.622 and 0.604, respectively (Fig. 2A,B). At the selected threshold, 26 of the 50 cases were classified as the HR group in test set. The sensitivity was 75.0% and the specificity was 63.3%. The F1 score was 0.6522 and the accuracy was 68%. The AUC in the training, test sets were 0.796, 0.763, respectively (Fig. 2C,D).

**Table 1:** Classification performance of the model.

	AUC	F1	PPV	NPV	Sensitivity	Specificity	Accuracy
Patch-level performance							
Training set (n = 13,368)	0.622	0.6332	0.5732	0.6051	0.7072	0.4602	0.5852
Test set (n = 5820)	0.604	0.6300	0.5422	0.6335	0.7518	0.4033	0.5722
Case-level performance							
Training set (n = 114)	0.796	0.6526	0.6596	0.7463	0.6458	0.7576	0.7105
Test set (n = 50)	0.763	0.6522	0.5769	0.7917	0.7500	0.6333	0.6800

*PPV* positive predictive value, *NPV* negative predictive value.

**Figure 3:**
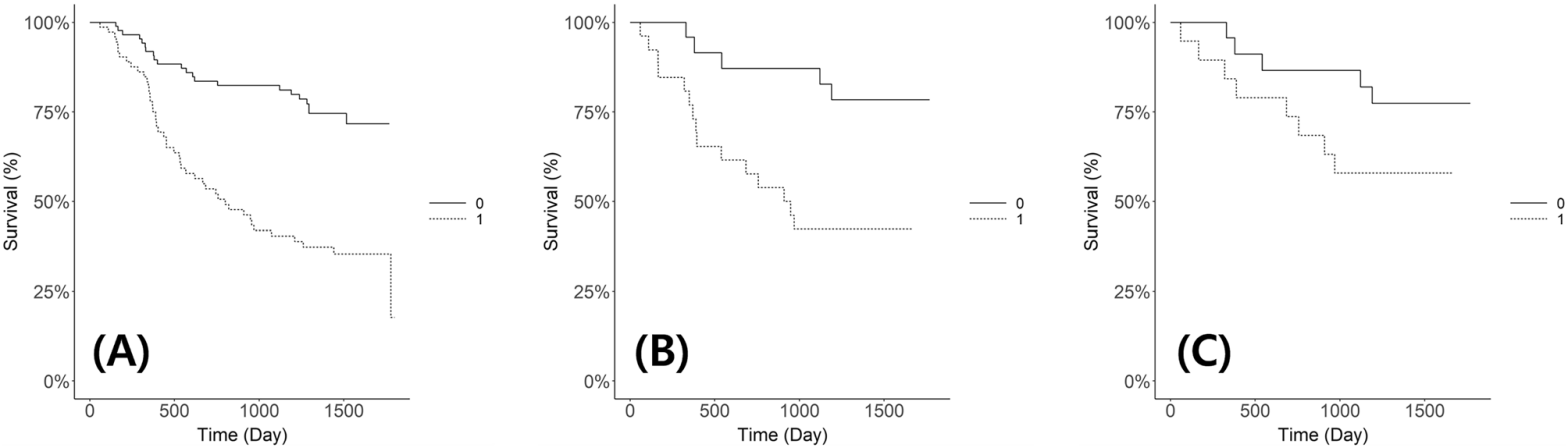
Kaplan–Meier estimation of recurrence-free survival. (**A**) General group, (**B**) testing set, (**C**) stages I-II in testing set.

The predicted HR groups were significantly associated with shorter RFS, even when the data were confined to stage I-II cases (Fig. 3A–C). The mean (± standard deviation [SD]) RFS was significantly shorter in the HR group (*p* < 0.001): HR group, 855.71 days (± 547.83), LR group, 1178.57 days (± 521.26). The mean overall survival (OS) was also shorter in the HR group, but the difference was not significant (*p* = 0.143).

## Histopathologic features according to risk group and recurrence

Histopathologic comparisons between the HR and LR group are summarized in Table 2. The tumor invasion size was larger in the HR group (*p* < 0.001). The proportion of the predominant histologic type was different between the groups (*p* < 0.001). Cases in which lepidic, acinar and papillary types were predominant, considered well to moderately differentiated histologic subtypes^27^, were more likely to be assigned to the LR group. In contrast, solid, micropapillary, mucinous and cribriform-predominant cases were only observed in the HR group. IASLC grades of the tumors were higher in the HR group (*p* < 0.001). The HR group had a higher proportion of PD and CGP components (*p* < 0.001, both). Necrosis, STAS, pleural invasion and lymphovascular invasion (LVI) were more common in the HR group (*p* < 0.001 for all comparisons, except STAS’s p = 0.003). pT, pN and stage group tended to be higher in the HR group (*p* < 0.001 for all comparisons).

**Table 2:** Clinicopathological characteristics of patients according to the model-based risk group.

Variables	Total	Low-risk (n = 91)	High-risk (n = 73)	*p* value
Age, year				
Mean (SD)	62.84 (10.67)	63.55 (10.65)	61.94 (10.69)	0.340
Sex				
Male	91 (55.5%)	45 (49.5%)	46 (63.0%)	0.114
Female	73 (44.5%)	46 (50.5%)	27 (37.0%)	
RFS, day				
Mean (SD)	1034.86 (555.42)	1178.57 (521.26)	855.71 (547.83)	< 0.001
OS, day				
Mean (SD)	1292.13 (483.20)	1341.74 (458.93)	1230.29 (508.24)	0.143
Tumor invasion size				
Mean (SD)	3.19 (1.76)	2.76 (1.43)	3.75 (1.97)	< 0.001
Predominant histologic type				
Lepidic	5 (3.0%)	5 (5.5%)	0 (0.0%)	< 0.001
Acinar	61 (37.2%)	38 (41.8%)	23 (31.5%)	
Papillary	43 (26.2%)	30 (33.0%)	13 (17.8%)	
Solid	33 (20.1%)	8 (8.8%)	25 (34.2%)	
Micropapillary	4 (2.4%)	2 (2.2%)	2 (2.7%)	
Cribriform	4 (2.4%)	1 (1.1%)	3 (4.1%)	
Mucinous	14 (8.5%)	7 (7.7%)	7 (9.6%)	
IASLC grade				
1	7 (4.3%)	6 (6.6%)	1 (1.4%)	< 0.001
2	51 (31.1%)	45 (49.5%)	6 (8.2%)	
3	106 (64.6%)	40 (44.0%)	66 (90.4%)	
PD component, %				
Mean (SD)	49.09 (38.60)	30.23 (34.66)	72.60 (29.34)	< 0.001
CGP component, %				
Mean (SD)	19.49 (25.16)	14.12 (20.93)	26.19 (28.34)	< 0.001
Necrosis				
Absent	100 (61.0%)	71 (78.0%)	29 (39.7%)	< 0.001
Present	64 (39.0%)	20 (22.0%)	44 (60.3%)	
STAS				
Absent	50 (30.5%)	37 (40.7%)	13 (17.8%)	0.003
Present	114 (69.5%)	54 (59.3%)	60 (82.2%)	
Pleural invasion				
Absent	105 (64.0%)	70 (76.9%)	35 (47.9%)	0.001
PL1	27 (16.5%)	10 (11.0%)	17 (23.3%)	
PL2	19 (11.6%)	8 (8.8%)	11 (15.1%)	
PL3	13 (7.9%)	3 (3.3%)	10 (13.7%)	
Lymphovascular invasion				
Absent	84 (51.2%)	62 (68.1%)	22 (30.1%)	< 0.001
Present	80 (48.8%)	29 (31.9%)	51 (69.9%)	
pT stage				
pT1	70 (42.7%)	52 (57.1%)	18 (24.7%)	< 0.001
pT2	58 (35.4%)	26 (28.6%)	32 (43.8%)	
pT3	26 (15.9%)	10 (11.0%)	16 (21.9%)	
pT4	10 (6.1%)	3 (3.3%)	7 (9.6%)	
pN stage				
pN0	117 (71.8%)	75 (83.3%)	42 (57.5%)	< 0.001
pN1	17 (10.4%)	3 (3.3%)	14 (19.2%)	
pN2	29 (17.8%)	12 (13.3%)	17 (23.3%)	
Stage group				
I	93 (56.7%)	64 (70.3%)	29 (39.7%)	< 0.001
II	33 (20.1%)	13 (14.3%)	20 (27.4%)	
III	36 (22.0%)	14 (15.4%)	22 (30.1%)	
IV	2 (1.2%)	0 (0.0%)	2 (2.7%)	

*SD* standard deviation, *IASLC* International Association for the Study of Lung Cancer, *PD* poorly differentiated, *CGP* complex glandular pattern, *STAS* tumor spread through air spaces, *PL* level of pleural invasion (PL1, visceral pleural elastic layer; PL2, visceral pleural surface; PL3, parietal pleura and/or chest wall).

**Figure 4:**
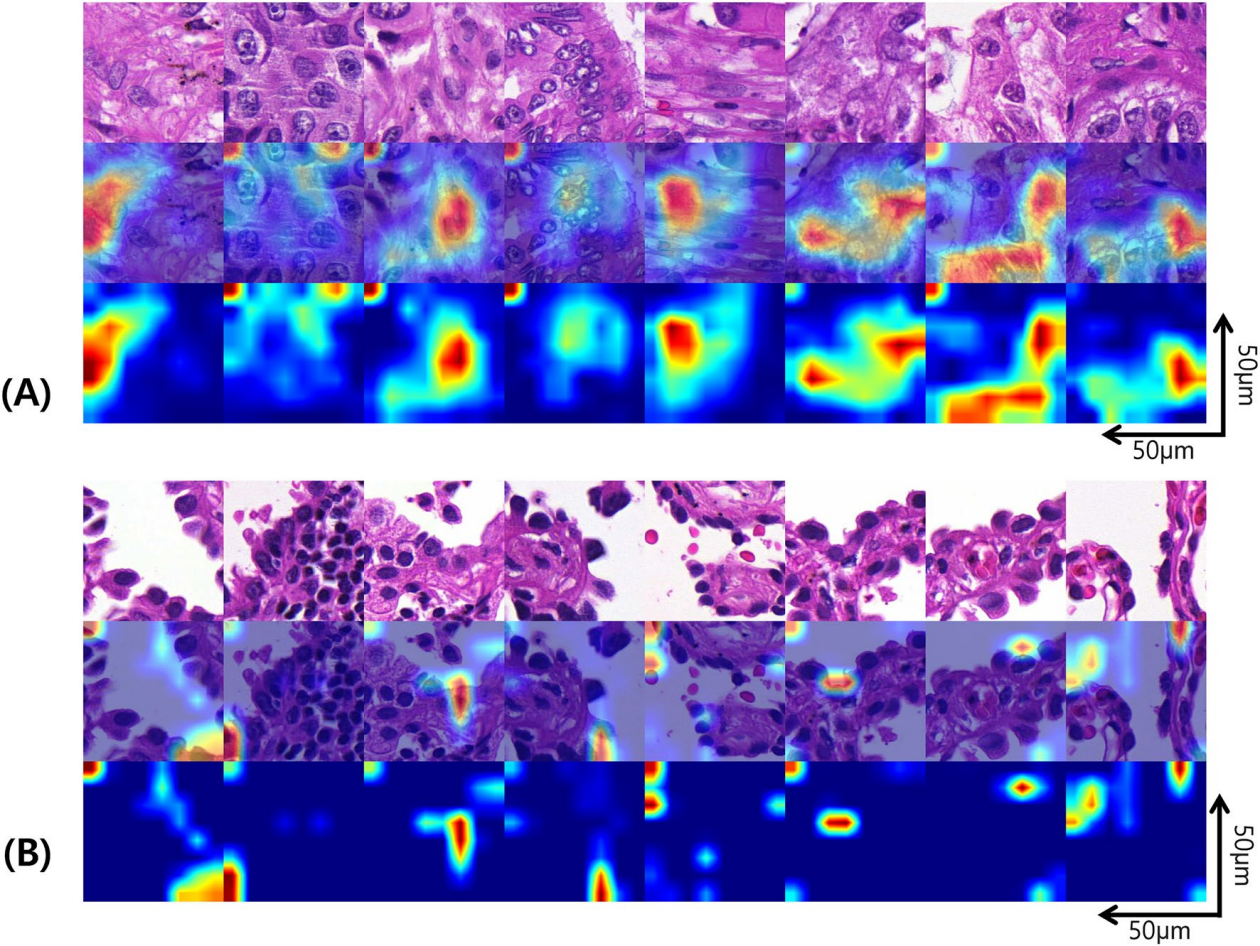
Class activation map of representative image patches. (**A**) Patches with the highest risk, (**B**) patches with the lowest risk.

**Figure 5:**
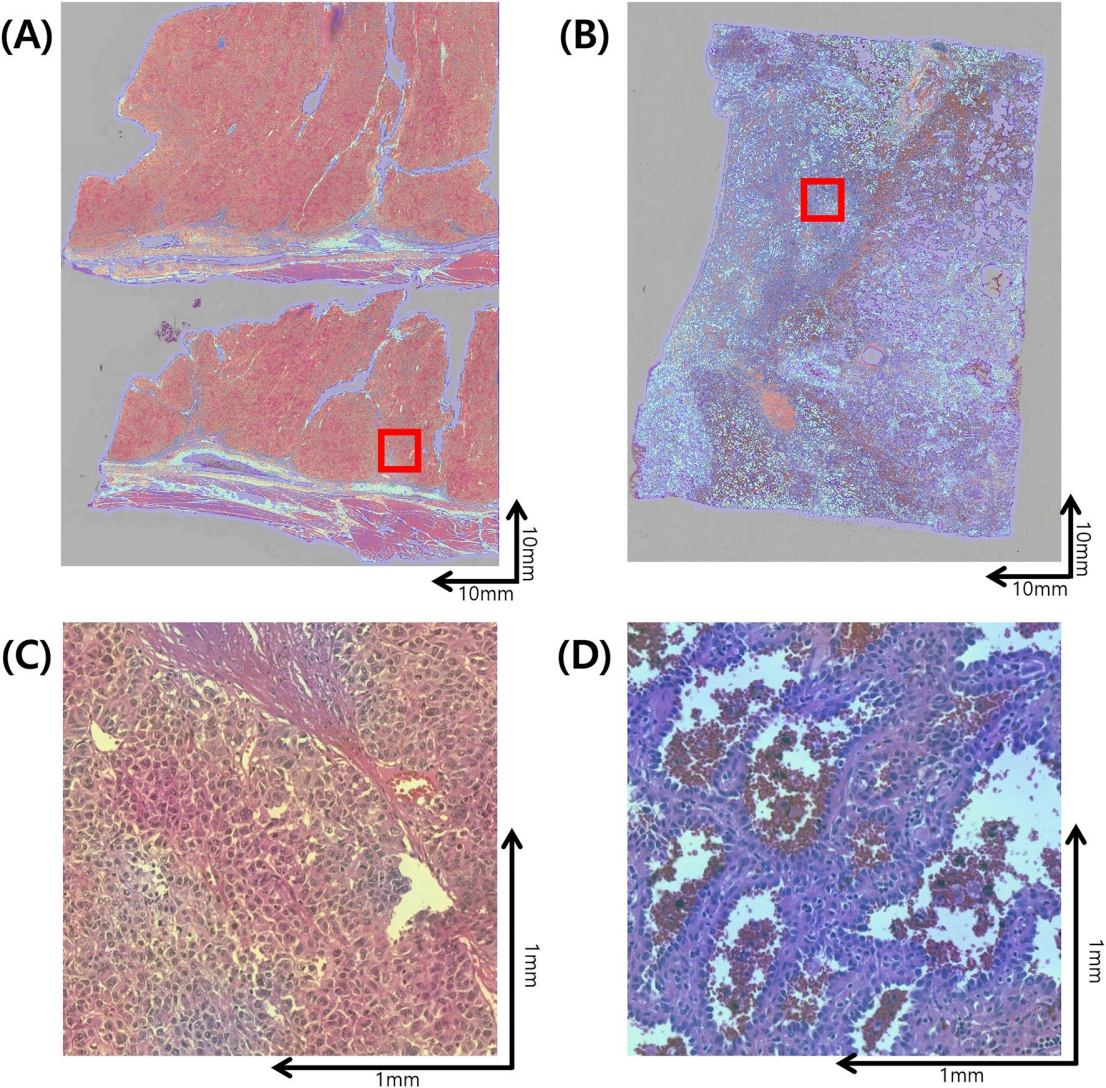
Heatmaps indicating tumor probability in whole slide images. (**A**) Whole slide image with the highest tumor probability classification. (**B**) Whole slide image with the lowest tumor probability classification. (**C**) Magnified image from slide (**A**) highlighting details. (**D**) Magnified image from slide (**B**) highlighting details.

Class activation maps (CAMs) shown in Fig. 4 display representative image patches with the highest risk (Fig. 4A) and the lowest risk (Fig. 4B). Representative LR patches were composed of relatively monotonous cells with lepidic or papillary growth patterns, while the HR patches had tumor cells with pleomorphic nuclei and complex structures. At the case level, The WSIs classified under the HR group often exhibit pronounced cellular pleomorphism, solid structures, and overall poor histological differentiation (Fig. 5A,C). On the other hand, WSIs classified under the LR group predominantly include well-differentiated histologic features with minimal tumor cell pleomorphism, displaying lepidic patterns as shown in Fig. 5B,D.

Additionally, we compared histologic features between patients grouped by their status of actual tumor recurrence. These results are summarized in Supplementary Table S2. The mean tumor invasion size and proportions of PD and CGP components were significantly higher in the recurrence group. IASLC grade, necrosis, STAS, pleural invasion, LVI, pT, pN and stage group were significantly higher in the recurrence group. On the other hand, a predominant histologic type was not significantly associated with recurrence (*p* = 0.923), validating the performance of the IASLC grade.

### Association with genomic alterations

NGS data from 163 cases were retrieved and the results are summarized in Table 3. Mutations in four genes were found in a significant number of patients: *CDKN2A*, *TP53*, *KRAS* and *EGFR*. The HR group was significantly associated with *TP53* alterations (*p* = 0.007) and in line with the model prediction, *TP53* alteration was significantly associated with cases of actual recurrence (*p* < 0.001). *ALK* translocation was found in 2 cases, all of which were assigned to the HR group.

**Table 3:** Genomic alterations according to the model-based risk group and recurrence.

Genomic alterations	Low-risk (n = 91)	High-risk (n = 73)	*p* value
*CDKN2A* alteration			
Absent	77 (85.6%)	58 (79.5%)	0.413
Present	13 (14.4%)	15 (20.5%)	
*TP53* alteration			
Absent	51 (56.7%)	25 (34.2%)	0.007
Present	39 (43.3%)	48 (65.8%)	
*KRAS* mutation			
Absent	78 (86.7%)	63 (86.3%)	1.000
Present	12 (13.3%)	10 (13.7%)	
*EGFR* mutation			
Absent	30 (33.3%)	34 (46.6%)	0.119
Present	60 (66.7%)	39 (53.4%)	
*ALK* translocation			
Absent	90 (100.0%)	71 (97.3%)	0.387
Present	0 (0.0%)	2 (2.7%)	

Genomic alterations	No recurrence (n = 96)	Recurrence (n = 68)	*p* value
*CDKN2A* alteration			
Absent	79 (82.3%)	56 (83.6%)	0.997
Present	17 (17.7%)	11 (16.4%)	
*TP53* alteration			
Absent	56 (58.3%)	20 (29.9%)	< 0.001
Present	40 (41.7%)	47 (70.1%)	
*KRAS* mutation			
Absent	84 (87.5%)	57 (85.1%)	0.831
Present	12 (12.5%)	10 (14.9%)	
*EGFR* mutation			
Absent	39 (40.6%)	25 (37.3%)	0.793
Present	57 (59.4%)	42 (62.7%)	
*ALK* translocation			
Absent	95 (99.0%)	66 (98.5%)	1.000
Present	1 (1.0%)	1 (1.5%)	

### Clinical and histopathological characteristics of stage I–II cases

Stage I–II cases were analyzed with more attention because this model could have a significant beneficial impact on these patients by guiding the selection of their adjuvant treatment. Stage I–II patients comprised 125 of the 164 cases (76.2%). Clinical and histopathological comparisons of the Stage I–II patients, when grouped by the model-based risk group and by actual recurrence status, revealed results similar to those of the all patients (Stages I–IV). Among the testing set data, 42 of 50 cases (84.0%) were Stages I–II and the HR group exhibited a significantly shorter RFS (Fig. 3C), validating its predictive performance in early-stage LUAD patients. OS was not significantly different. The detailed clinical and histopathological comparison data of this group are provided in Supplementary Tables S3 and S4.

## Discussion

In this study, we developed a model to predict the risk of recurrence of LUAD by DL-based image analysis. This classification model showed good performance with high sensitivity, implying its potential usefulness as a screening tool. The model revealed an AUC of 0.763 in the testing set, which is better performance to the IASLC grade (an AUC of 0.690)^9^. The predicted risk groups were strongly correlated with histopathological features and several genetic mutations. Clinicopathologic results for stage I–II cases were virtually the same as those of the general group.

Pathological research typically sees strong AI model performance in areas where histological differences are easily recognized by pathologists. Unfortunately, in the case of LUAD, histological characteristics are diverse and complex, making it challenging for pathologists to discern differences easily. The present study was aimed an exploratory effort to determine if an AI model can successfully identify histological differences between recurrence and non-recurrence in early-stage lung cancer cases with partial resection—an unresolved challenge for pathologists. This study demonstrated the AI model's potential to predict recurrence in partially resected lung tissue, marking a significant achievement. If efforts to introduce more advanced models based on this research and develop algorithms explaining the model's decisions are attempted in future studies, it is anticipated that identifying patients in need of closer monitoring will become possible, leading to improved patient survival.

Lung cancer has various histological types such as LUAD, squamous cell carcinoma, and small cell lung cancer^28^. Squamous cell carcinoma primarily originates in the central part of the lung, and when surgery is feasible, lobectomy is commonly performed. Therefore, this type of tumor is generally not considered a candidate for partial resection. In the case of small cell lung cancer, which also typically arises in the central region of the lung, standard treatments include radiation therapy or chemotherapy. LUAD, the most common histological subtype at 38.5%, is experiencing a significant increase in incidence and is the most common subtype for which partial resection is performed^29^. Considering the significant histological differences among these three types, we chose adenocarcinoma as the focus of our study to create a meaningful model, specifically predicting tumor recurrence after partial resection, for clinical practice. We anticipated that creating a model encompassing all three histological subtypes would be challenging due to their distinct characteristics. Additionally, considering the target application of the model, we judged that including all three tumors from a clinical perspective would not be suitable.

The model’s output reflects histopathological features known to be associated with the tumor biology. The structural pattern is currently the most important factor in the histological subtyping of LUAD^9^. The HR group showed not only significantly higher proportions of PD and CGP components, but also more complex pattern in representative image patches than the LR group. Enlarged and pleomorphic nuclei in the HR patches are consistent with previous studies, which showed that nuclear size is more significantly associated with the prognosis than the nuclear to cytoplasm ratio (N/C ratio) in LUAD^30,31^. In addition, we showed various histopathologic parameters like STAS, pleural invasion, and LVI were significantly associated with the HR group, although they might not be reflected in the patch-level evaluation of the model because they are usually observed in sparsely scattered areas around the tumor border. It suggests that the HR group has aggressive phenotype.

Detection of genomic alterations of LUAD by the DL-based model has been successful in previous studies^19,32^. Our study also showed biological feature reflected by the model was its association with *TP53* alterations^33,34^. *TP53* are tumor suppressor genes, and its mutations are known to be associated with tumor progression and poor prognosis^33,34^. From the perspective of the tumor immune microenvironment, *TP53* alterations in LUAD have been reported to be associated with high infiltration of M0 macrophages and an immunosuppressive environment, along with *KRAS* mutations^35,36^. These cases may have a high potential for the effectiveness of immune checkpoint inhibitors (ICIs). If the present model is tuned to more accurately predict *TP53* gene mutations, it could serve as a valuable screening test for selectively applying adjuvant ICI treatment, such as PD-L1 inhibitors in LUAD patients who have undergone partial resection at the early stages of *TP53* gene alterations.

This study and previous studies^17,18^ demonstrated the potential of DL-based risk prediction of LUAD using histopathological images. This study lies in the utilization of actual patient data, serving as the direct application target for the developed model, employing various DL architectures, and notably enhancing predictive power through the application of MIL. Moreover, the study not only confirmed the model's emphasis on distinguishing HR and LR recurrence groups by comparing detailed interpretations of a specialized pulmonary pathologist and various cancer genetic variations but also elucidated the model's specific interpretability by highlighting its correlation with various histopathological findings and genetic changes currently crucial in LUAD pathology interpretation. The results from DL-based models were good but still suboptimal for clinical practice use. Insufficient data size, heterogenous histology of LUAD, confounding elements including epithelioid macrophages or lack of optimized DL architecture could limit the performance of histopathologic models. However, a study from the IASLC group showed that the power of histologic characteristics as a tool for prognosis prediction is limited^9^. A critical improvement could be achieved by a multidisciplinary approach, including clinical and genetic data along with histological features. Several studies have attempted such an approach^37,38^, but they did not fully integrate pathological images into their models. Further studies are warranted.

In conclusion, the DL model showed good performance in recurrence prediction by analyzing histopathological images. The predicted risk group was associated with aggressive biological features. The model can provide useful information for the risk stratification and the selection of treatment of LUAD.

### Supplementary Information

Supplementary Information.

## Data Availability

Data will be made available on request to corresponding author and with the permission of the institutional review board of Asan Medical Center.
