# Supplementary material for: A new model using deep learning to predict recurrence after surgical resection of lung adenocarcinoma
Source: Sci Rep. 2024 Mar 16;14:6366. doi: 10.1038/s41598-024-56867-9 (PMC10944489; doi:10.1038/s41598-024-56867-9)
Supplement: Supplementary file 1 — Supplementary Information. [file 41598_2024_56867_MOESM1_ESM.docx]

**Supplementary Table S1. Validation error in cross validation in each deep learning model**

|  | efficientNet_b2 | densenet201 | resnet152 |
| --- | --- | --- | --- |
| CV1 | 53.87 | 52.51 | 53.21 |
| CV2 | 55.70 | 53.02 | 54.46 |
| CV3 | 56.33 | 55.98 | 54.88 |
| CV4 | 51.18 | 52.07 | 51.33 |
| CV5 | 58.12 | 57.03 | 55.04 |
| Average | 55.04 | 54.12 | 53.78 |

CV, cross validation

**Supplementary Table S2. Histopathological characteristics of patients according to recurrence**

| Variables |  | Total | No recurrence  (n = 96) | Recurrence  (n = 68) | *P*-value |
| --- | --- | --- | --- | --- | --- |
| Age, year | Mean (SD) | 62.84 (10.67) | 64.04 (9.76) | 61.13 (11.70) | 0.085 |
| Sex | Male | 91 (55.5%) | 52 (54.2%) | 39 ( 57.4%) | 0.806 |
|  | Female | 73 (44.5%) | 44 (45.8%) | 29 ( 42.6%) |  |
| RFS, day | Mean (SD) | 1034.86 (555.42) | 1343.48 (429.42) | 599.16 (399.90) | <0.001 |
| OS, day | Mean (SD) | 1292.13 (483.20) | 1343.48 (429.42) | 1219.63 (545.38) | 0.106 |
| Tumor invasion size | Mean (SD) | 3.19 (1.76) | 2.78 (1.52) | 3.78 (1.92) | <0.001 |
| Predominant histologic type | Lepidic | 5 (3.0%) | 4 (4.2%) | 1 (1.5%) | 0.923 |
|  | Acinar | 61 (37.2%) | 35 (36.5%) | 26 (38.2%) |  |
|  | Papillary | 43 (26.2%) | 25 (26.0%) | 18 (26.5%) |  |
|  | Solid | 33 (20.1%) | 18 (18.8%) | 15 (22.1%) |  |
|  | Micropapillary | 4 (2.4%) | 2 (2.1%) | 2 (2.9 %) |  |
|  | Cribriform | 4 (2.4%) | 3 (3.1%) | 1 (1.5%) |  |
|  | Mucinous | 14 (8.5%) | 9 (9.4%) | 5 (7.4%) |  |
| IASLC grade | 1 | 7 (4.3%) | 6 (6.2%) | 1 (1.5%) | <0.001 |
|  | 2 | 51 (31.1%) | 43 (44.8%) | 8 (11.8%) |  |
|  | 3 | 106 (64.6%) | 47 (49.0%) | 59 (86.8%) |  |
| PD component, % | Mean (SD) | 49.09 (38.60) | 37.53 (39.13) | 65.41 (31.49) | <0.001 |
| CGP component, % | Mean (SD) | 19.49 (25.16) | 12.23 (20.86) | 29.75 (27.21) | <0.001 |
| Necrosis | Absent | 100 (61.0%) | 71 (74.0%) | 29 (42.6%) | <0.001 |
|  | Present | 64 (39.0%) | 25 (26.0%) | 39 (57.4%) |  |
| STAS | Absent | 50 (30.5%) | 37 (58.5%) | 13 (19.1%) | 0.015 |
|  | Present | 114 (69.5%) | 59 (61.5%) | 55 (80.9%) |  |
| Pleural invasion | Absent | 105 (64.0%) | 74 (77.1%) | 31 (45.6%) | <0.001 |
|  | PL1 | 27 (16.5%) | 12 (12.5%) | 15 (22.1%) |  |
|  | PL2 | 19 (11.6%) | 6 (6.3%) | 13 (19.1%) |  |
|  | PL3 | 13 (7.9%) | 4 (4.2%) | 9 (13.2%) |  |
| Lymphovascular invasion | Absent | 84 (51.2%) | 65 (67.7%) | 19 (27.9%) | <0.001 |
|  | Present | 80 (48.8%) | 31 (32.3%) | 49 (72.1%) |  |
| pT stage | pT1 | 70 (42.7%) | 54 (56.2%) | 16 (23.5%) | <0.001 |
|  | pT2 | 58 (35.4%) | 30 (31.2%) | 28 (41.2%) |  |
|  | pT3 | 26 (15.9%) | 9 (9.4%) | 17 (25.0%) |  |
|  | pT4 | 10 (6.1%) | 3 (3.1%) | 7 (10.3%) |  |
| pN stage | pN0 | 117 (71.8%) | 80 (84.2%) | 37 (54.4%) | <0.001 |
|  | pN1 | 17 (10.4%) | 6 (6.3%) | 11 (16.2%) |  |
|  | pN2 | 29 (17.8%) | 9 (9.5%) | 20 (29.4%) |  |
| Stage group | I | 93 (56.7%) | 69 (71.8%) | 24 (35.3%) | <0.001 |
|  | II | 33 (20.1%) | 15 (15.6%) | 18 (26.5%) |  |
|  | III | 36 (22.0%) | 11 (11.5%) | 25 (36.8%) |  |
|  | IV | 2 (1.2%) | 1 (1.0%) | 1 (1.5%) |  |

SD, standard deviation; IASLC, International Association for the Study of Lung Cancer; PD, poorly differentiated; CGP, complex glandular pattern; STAS, tumor spread through air spaces; PL, level of pleural invasion (PL1, visceral pleural elastic layer; PL2, visceral pleural surface; PL3, parietal pleura and/or chest wall)

**Supplementary Table S3. Clinicopathological characteristics of patients with Stage I-II according to the model-based risk group**

| Variables |  | Total | Low-risk  (n = 76) | High-risk  (n = 49) | *P*-value |
| --- | --- | --- | --- | --- | --- |
| Age, year | Mean (SD) | 63.60 (10.42) | 64.65 (9.73) | 61.98 (11.32) | 0.164 |
| Sex | Male | 69 (55.2%) | 41 ( 53.9 %) | 28 ( 57.1 %) | 0.868 |
|  | Female | 56 (44.8%) | 35 ( 46.1 %) | 21 ( 42.9 %) |  |
| RFS, day | Mean (SD) | 1129.46 (519.63) | 1223.70 (496.27) | 983.31 (526.18) | 0.011 |
| OS, day | Mean (SD) | 1344.60 (452.20) | 1377.75 (433.43) | 1293.18 (479.89) | 0.309 |
| Tumor invasion size | Mean (SD) | 2.80 (1.27) | 2.63 (1.28) | 3.07 (1.22) | 0.057 |
| Necrosis | Absent | 88 (70.4%) | 62 (81.6 %) | 26 ( 53.1 %) | 0.001 |
|  | Present | 37 (29.6%) | 14 (18.4 %) | 23 (46.9 %) |  |
| Predominant histologic type | Lepidic | 5 (4.0%) | 5 (6.6 %) | 0 (0.0 %) | <0.001 |
|  | Acinar | 49 (39.2) | \| 27 (35.5 %) \| 9 (18.4 %) \| \| --- \| --- \| | 15 (30.6 %) |  |
|  | Papillary | 36 (28.8%) | 27 (35.5 %) | 9 (18.4 %) |  |
|  | Solid | 23 (18.4%) | 5 (6.6 %) | 18 (36.7 %) |  |
|  | Micropapillary | 1 (0.8%) | 0 (0.0 %) | 1 (2.0 %) |  |
|  | Cribriform | 2 (1.6%) | 0 (0.0 %) | 2 (4.1 %) |  |
|  | Mucinous | 9 (7.2%) | 5 (6.6 %) | 4 (8.2 %) |  |
| IASLC grade | 1 | 7 (5.6%) | 6 (7.9 %) | 1 (2.0 %) | <0.001 |
|  | 2 | 47 (37.6%) | 42 (55.3 %) | 5 (10.2 %) |  |
|  | 3 | 71 (56.8%) | 28 (36.8 %) | 43 (87.8 %) |  |
| PD component, % | Mean (SD) | 41.82 (38.31) | 24.15 (31.39) | 69.22 (31.48) | <0.001 |
| CGP component, % | Mean (SD) | 17.82 (24.43) | 12.86 (20.89) | 25.51 (27.58) | <0.001 |
| STAS | Absent | 44 (35.2%) | 35 (46.7 %) | 9 (18.4 %) | 0.002 |
|  | Present | 81 (64.8%) | 40 (53.3 %) | 40 (81.6 %) |  |
| Pleural invasion | Absent | 93 (74.4%) | 62 (81.6 %) | 31 (63.3 %) | 0.152 |
|  | PL1 | 16 (12.8%) | 7 (9.2 %) | 9 (18.4 %) |  |
|  | PL2 | 11 (8.8%) | 5 (6.6 %) | 6 (12.2 %) |  |
|  | PL3 | 5 (4.0%) | 2 (2.6 %) | 3 (6.1 %) |  |
| Lymphovascular invasion | Absent | 80 (64.0%) | 58 (76.3 %) | 22 (44.9 %) | <0.001 |
|  | Present | 45 (36.0%) | 18 (23.7 %) | 27 (55.1 %) |  |
| pT stage | pT1 | 63 (50.4%) | 46 (60.5 %) | 17 (34.7 %) | 0.014 |
|  | pT2 | 48 (38.4%) | 22 (28.9 %) | 26 (53.1 %) |  |
|  | pT3 | 14 (11.2%) | 8 (10.5 %) | 6 (12.2 %) |  |
| pN stage | pN0 | 113 (90.4%) | 74 (97.4 %) | 39 (79.6 %) | 0.003 |
|  | pN1 | 12 (9.6%) | 2 (2.6 %) | 10 (20.4 %) |  |
| Stage group | I | 92 (73.6%) | 63 (82.9 %) | 29 (59.2 %) | 0.006 |
|  | II | 33 (76.4%) | 13 (17.1 %) | 20 (40.8 %) |  |

SD, standard deviation; IASLC, International Association for the Study of Lung Cancer; PD, poorly differentiated; CGP, complex glandular pattern; STAS, tumor spread through air spaces; PL, level of pleural invasion (PL1, visceral pleural elastic layer; PL2, visceral pleural surface; PL3, parietal pleura and/or chest wall)

**Supplemental Table S4. Clinicopathological characteristics of patients with Stage I-II according to the recurrence**

| Variables |  | Total | No recurrence  (n = 83) | Recurrence  (n = 42) | *P*-value |
| --- | --- | --- | --- | --- | --- |
| Age, year | Mean (SD) | 63.60 (10.4) | 64.51 (9.81) | 61.81 (11.45) | 0.173 |
| Sex | Male | 69 (55.2%) | 45 (54.2%) | 24 (57.1%) | 0.904 |
|  | Female | 56 (44.8%) | 38 (45.8%) | 18 (42.9%) |  |
| RFS, day | Mean (SD) | 1129.464 (519.63) | 1371.80 (385.18) | 650.57 (407.55) | <0.001 |
| OS, day | Mean (SD) | 1344.60 (452.20) | 1371.80 (385.18) | 1290.86 (563.27) | 0.347 |
| Tumor invasion size | Mean (SD) | 2.80 (1.27) | 2.55 (1.24) | 3.28 (1.19) | 0.002 |
| Necrosis | Absent | 88 (70.4%) | 66 (79.5%) | 22 (52.4%) | 0.003 |
|  | Present | 37 (29.6%) | 17 (20.5%) | 20 (47.6%) |  |
| Predominant histologic type | Lepidic | 5 (4.0 %) | 4 (4.8%) | 1 (2.4%) | 0.835 |
|  | Acinar | 49 (39.2%) | 31 (37.3%) | 18 (42.9%) |  |
|  | Papillary | 36 (28.8%) | 23 (27.7%) | 13 (31.0%) |  |
|  | Solid | 23 (18.4%) | 15 (18.1%) | 8 (19.0%) |  |
|  | Micropapillary | 1 (0.8%) | 1 (1.2%) | 0 (0.0%) |  |
|  | Cribriform | 2 (1.6%) | 2 (2.4%) | 0 (0.0%) |  |
|  | Mucinous | 9 (7.2%) | 7 (8.4%) | 2 (4.8%) |  |
| IASLC grade | 1 | 7 (5.6%) | 6 (7.2%) | 1 (2.4%) | <0.001 |
|  | 2 | 47 (37.6%) | 40 (48.2%) | 7 (16.7%) |  |
|  | 3 | 71 (56.8%) | 37 (44.6%) | 34 (81.0%) |  |
| PD component, % | Mean (SD) | 41.82 (38.31) | 33.40 (37.87) | 58.45 (33.83) | <0.001 |
| CGP component, % | Mean (SD) | 17.82 (24.43) | 10.31 (18.59) | 32.64 (27.84) | <0.001 |
| STAS | Absent | 44 (35.2%) | 36 (43.4%) | 8 (19.0%) | 0.016 |
|  | Present | 81 (64.8%) | 47 (56.6%) | 34 (81.0%) |  |
| Pleural invasion | Absent | 93 (74.4%) | 69 (83.1%) | 24 (57.1%) | 0.007 |
|  | PL1 | 16 (12.8%) | 8 (9.6%) | 8 (19.0%) |  |
|  | PL2 | 11 (8.8%) | 3 (3.6%) | 8 (19.0%) |  |
|  | PL3 | 5 (4.0%) | 3 (3.6%) | 2 (4.8%) |  |
| Lymphovascular invasion | Absent | 80 (64.0%) | 62 (74.7%) | 18 (42.9%) | <0.001 |
|  | Present | 45 (36.0%) | 21 (25.3%) | 24 (57.1%) |  |
| pT stage | pT1 | 63 (50.4%) | 50 (60.2%) | 13 (31.0%) | 0.008 |
|  | pT2 | 48 (38.4%) | 26 (31.3%) | 22 (52.4%) |  |
|  | pT3 | 14 (11.2%) | 7 (8.4 %) | 7 (16.7%) |  |
| pN stage | pN0 | 113 (90.4%) | 79 (95.2%) | 34 (81.0%) | 0.026 |
|  | pN1 | 12 (9.6%) | 4 (4.8%) | 8 (19.0%) |  |
| Stage group | I | 92 (73.6%) | 68 (81.9%) | 24 (57.1%) | 0.006 |
|  | II | 33 (26.4%) | 15 (18.1 %) | 18 (42.9%) |  |

SD, standard deviation; IASLC, International Association for the Study of Lung Cancer; PD, poorly differentiated; CGP, complex glandular pattern; STAS, tumor spread through air spaces; PL, level of pleural invasion (PL1, visceral pleural elastic layer; PL2, visceral pleural surface; PL3, parietal pleura and/or chest wall)
